# Effects of a Web-Based Tailored Intervention to Reduce Alcohol Consumption in Adults: Randomized Controlled Trial

**DOI:** 10.2196/jmir.2568

**Published:** 2013-09-17

**Authors:** Daniela N Schulz, Math JJM Candel, Stef PJ Kremers, Dominique A Reinwand, Astrid Jander, Hein de Vries

**Affiliations:** ^1^CAPHRI School for Public Health and Primary CareDepartment of Health PromotionMaastricht UniversityMaastrichtNetherlands; ^2^CAPHRI School for Public Health and Primary CareDepartment of Methodology and StatisticsMaastricht UniversityMaastrichtNetherlands; ^3^Nutrition and Toxicology Research Institute Maastricht (NUTRIM)Department of Health PromotionMaastricht UniversityMaastrichtNetherlands; ^4^Jacobs University Bremen gGmbHJacobs Center on Lifelong Learning and Institutional DevelopmentBremenGermany

**Keywords:** alcohol intake, adults, eHealth, computer tailoring, Web-based intervention, tailoring methods, effectiveness

## Abstract

**Background:**

Web-based tailored interventions provide users with information that is adapted to their individual characteristics and needs. Randomized controlled trials assessing the effects of tailored alcohol self-help programs among adults are scarce. Furthermore, it is a challenge to develop programs that can hold respondents’ attention in online interventions.

**Objective:**

To assess whether a 3-session, Web-based tailored intervention is effective in reducing alcohol intake in high-risk adult drinkers and to compare 2 computer-tailoring feedback strategies (alternating vs summative) on behavioral change, dropout, and appreciation of the program.

**Methods:**

A single-blind randomized controlled trial was conducted with an experimental group and a control group (N=448) in Germany in 2010-2011. Follow-up took place after 6 months. Drinking behavior, health status, motivational determinants, and demographics were assessed among participants recruited via an online access panel. The experimental group was divided into 2 subgroups. In the alternating condition (n=132), the tailored feedback was split into a series of messages discussing individual topics offered while the respondent was filling out the program. Participants in the summative condition (n=181) received all advice at once after having answered all questions. The actual texts were identical for both conditions. The control group (n=135) only filled in 3 questionnaires. To identify intervention effects, logistic and linear regression analyses were conducted among complete cases (n=197) and after using multiple imputation.

**Results:**

Among the complete cases (response rate: 197/448, 44.0%) who did not comply with the German national guideline for low-risk drinking at baseline, 21.1% of respondents in the experimental group complied after 6 months compared with 5.8% in the control group (effect size=0.42; OR 2.65, 95% CI 1.14-6.16, *P*=.02). The experimental group decreased by 3.9 drinks per week compared to 0.4 drinks per week in the control group, but this did not reach statistical significance (effect size=0.26; beta=−0.12, 95% CI −7.96 to 0.03, *P*=.05). Intention-to-treat analyses also indicated no statistically significant effect. Separate analyses of the 2 experimental subgroups showed no differences in intervention effects. The dropout rate during the first visit to the intervention website was significantly lower in the alternating condition than in the summative condition (OR 0.23, 95% CI 0.08-0.60, *P*=.003). Program appreciation was comparable for the 2 experimental groups.

**Conclusions:**

Complete case analyses revealed that Web-based tailored feedback can be an effective way to reduce alcohol intake among adults. However, this effect was not confirmed when applying multiple imputations. There was no indication that one of the tailoring strategies was more effective in lowering alcohol intake. Nevertheless, the lower attrition rates we found during the first visit suggest that the version of the intervention with alternating questions and advice may be preferred.

**Trial Registration:**

International Standard Randomized Controlled Trial Number (ISRCTN): 91623132; http://www.controlled-trials.com/ISRCTN91623132 (Archived by WebCite at http://www.webcitation.org/6J4QdhXeG).

## Introduction

Although the consumption of alcohol is associated with numerous negative consequences, such as cardiovascular disease, cancer, cirrhosis, neuropsychiatric disorders, traffic accidents, and reduced work productivity [1-3], high alcohol consumption is highly prevalent among adults worldwide [4-6]. Many people with unhealthy drinking patterns are not aware of their alcohol intake or the problems associated with this behavior [7,8]; others are aware, but do not seek care, help, or support [9-11] possibly out of fear, shame, or lack of time. The high prevalence of unhealthy drinkers and the low number of them who seek help underline the need for easily accessible and low-threshold interventions to encourage people to reduce their alcohol intake.

Web-based tailored interventions in which information is adapted to the user’s individual characteristics and needs to give them personally appropriate advice [12,13] have proved an effective tool to improve health-related behaviors. Various studies have reported favorable effects on lifestyle behaviors, such as increasing physical activity [14,15], increasing fruit and vegetable consumption and lowering saturated fat intake [16], and giving up smoking [17]. The main advantages of intervention programs providing tailored advice compared to nontailored materials are that they contain less unnecessary information and more attractive and relevant information [18,19], they are cost-effective [20], the tailored messages are more likely to be read, saved, printed out, remembered, and discussed with others [13,21-23], and tailored information is more effective for behavior change than generic messages [12,24,25].

To date, several studies of Web-based tailored alcohol interventions have been published, but randomized controlled trials among the general adult population using tailored self-help programs have been scarce [25-32]. Most previous studies were conducted among young people, especially among university or student populations [33-37]. These samples are not representative of the general population and may, for example, differ in motivation to change, reading level, computer and Internet access, and computer literacy [37]. Earlier studies reported that single-session, individually personalized feedback without therapeutic guidance can be an effective and cost-effective method to reduce alcohol consumption [38]. A recently published study of adult men using a single-session intervention in which respondents had to go to a laboratory to participate in an online 10-minute intervention reported only on short-term effects 1 month after the intervention [26].

Little research has been done to assess what elements work well in tailored interventions. The 5 criteria of Health Behavior Change treatment on the Internet (HBC-I)—advise, assist, assess, provide anticipatory guidance, and arrange follow-up—form essential, but not sufficient, elements that determine whether a program offers potential for behavior change [39]. Other feasible elements appear to be the use of tailoring strategies, such as normative, positive, and ipsative feedback, personal tone, and empathy [40]. Factors explaining the differences in effectiveness of programs include the number of contact/exposure moments, the use of theory, the layout, the communication channel, the length of the questionnaires, the amount of information given, and the depth of tailoring [12,41].

Although Internet-based programs have the potential to reach large numbers of people, various studies have pointed out that the actual use may be limited and that high rates of attrition are common [42-47]. To prevent early dropout and, thus, increase the effectiveness of a program, 2 different strategies could be used to hold respondents’ attention in online interventions. In the first strategy, questions and advice are given alternately, so that the respondents are rewarded while they are still filling in the questionnaires and are thereby motivated to continue. Such alternation might also enhance the attractiveness of the program. In the second strategy, advice is given in a more traditional way at the end of the session (ie, after the last question has been completed). This method may lead to postponement of dropout—provided that the questionnaires are not too long—because respondents have to wait until the end of the questionnaire before receiving tailored feedback. Yet, this method may also increase the risk that the participant becomes overwhelmed by the amount of information he or she receives all at once [48].

The objective of our study was twofold. First, we explored the overall effectiveness of a 3-session, Web-based, tailored alcohol intervention for unhealthy drinkers in the general adult population. Second, we compared the dropout rate, effectiveness, and user satisfaction of 2 kinds of feedback strategies (alternating vs summative).

## Methods

### Participants, Procedure, and Study Design

We conducted a randomized controlled trial (ISRCTN91623132) involving an experimental group and a waiting list control group, with a follow-up measurement after 6 months. The intervention, focusing on unhealthy drinkers in the general population, was conducted online in Germany in June 2010 to January 2011. Adult participants were recruited via an online access panel (ie, a register of a sample who expressed willingness to participate in online surveys and research studies) called respondi AG (place of business: Cologne, Germany). The sample received an email containing a link to either the intervention website (experimental group) or a Web-based alcohol questionnaire (control group). Randomization was carried out by a computer system. Two reminder messages in the form of emails were sent to individuals of the sample who did not respond to the first invitation. Incentives, in the form of bonus points that respondents could exchange for cash, a gift voucher, or a charitable donation were given to respondents who filled in the questionnaires completely. Informed consent was given during the registration process as a panel member in which the members gave permission to use their data for scientific research.

### Inclusion Criteria

The following inclusion criteria were established for this study: being a panel member of respondi; having computer/Internet literacy; having sufficient command of German; being 18 years or older; and having an unhealthy drinking pattern, which was defined as (1) not complying with the guideline recommending no more than 1 glass (women) or 2 glasses (men) of alcohol per day, (2) drinking on more than 5 days per week, (3) having a score higher than 7 on the Alcohol Use Disorders Identification Test (AUDIT) [49], or (4) currently trying to become pregnant, drinking alcohol while pregnant or breastfeeding (in relation to pregnancy), or trying to get one’s partner pregnant (for men).

### Intervention

**Figure 1:**
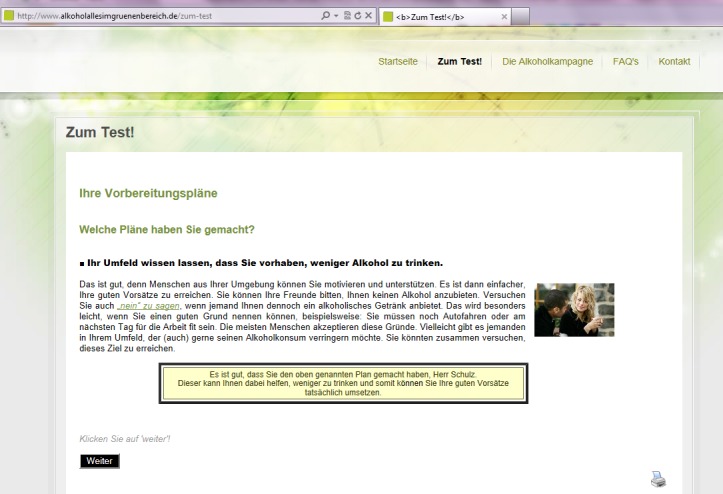
Screenshot of the intervention website, showing personal advice regarding preparatory plans.

The intervention program, called Alcohol-Everything Within the Limits?! (German: “Alkohol-Alles im grünen Bereich?!,” see Figure 1), is a Web-based, 3-session, tailored program targeting adult problem drinkers. The main aim of the intervention was to stimulate participants to lower their alcohol intake. The theoretical framework for the development of the intervention was the I-Change model [50]. This psychosocial model was chosen because it combines different models and integrates these in premotivational, motivational, and postmotivational phases, which is optimal for use in computer tailoring to support the process of behavioral change. The I-Change model builds on other psychosocial models, such as the Theory of Planned Behavior [51], Social Cognitive Theory [52], the Health Belief Model [53], and the Transtheoretical Model [54].

The personalized advice, which was presented immediately on the respondent’s computer screen, consisted of 5 parts, each focusing on a different psychosocial construct of the model (ie, knowledge, awareness, attitude, social influence, self-efficacy, and action planning). The first part of the program served as a starting point of the drinking behavior change process (premotivational phase) by addressing the concepts of knowledge and awareness: it gave information about the German alcohol guidelines, specifically, not drinking more than 1 (women) or 2 (men) standard drinks (ie, drinks containing 10 grams of alcohol) per day and having at least 2 alcohol-free days a week, and assessed whether respondents were meeting this guideline by using comparative/normative feedback. In addition, respondents’ scores were depicted graphically using a traffic light symbol (indicating whether they met, almost met, or did not meet the guidelines). To increase the respondent’s level of knowledge, the relation between alcohol and various diseases was explained, and information tailored to the respondent’s health status was given about alcohol and pregnancy, and about the possible influence of participants’ drinking behavior on their children (if applicable). The second part of the program offered personalized feedback concerning the perceived pros and cons of alcohol drinking as perceived by the respondent, with the goal of creating a positive attitude toward not drinking more than 1 (women) or 2 (men) alcoholic drinks per day. The third part explained the importance of social influence in a tailored message by focusing on the respondent’s partner, family, friends, and colleagues. In the fourth part, preparatory action plans were defined to prepare the intended behavioral change. The final part focused on self-efficacy and coping plans by identifying difficult situations and suggesting ways to cope with them. Personalized tips were given on how to deal with the perceived difficult situations to overcome potential barriers (postmotivational phase), and the situations and plans were summarized for individual respondents to help them remember these.

During the feedback moment after 3 months and the follow-up measurement after 6 months, participants of the experimental group again received personalized advice based on their previous scores for the psychosocial constructs. Additionally, ipsative feedback was given about the respondents’ alcohol intake by comparing the drinking score at the current visit with that at the last visit or visits. Feedback was given about potential change and all scores were illustrated in a graph to enable the respondent to monitor the total change process at a glance.

### Conditions

All study groups received identical questionnaires. After completing the third measurement, respondents in the waiting list control group were given the link to the intervention website where they could also receive personalized advice. The experimental condition was divided into 2 subgroups. The intervention website for these 2 subgroups offered the same feedback messages. At all 3 feedback moments (at baseline, after 3 months, and after 6 months), 1 experimental subgroup received questions and personal advice alternately (alternating condition) whereas respondents in the other experimental subgroup were given all personal advice at once after having answered all the questions (summative condition). In other words, in the alternating condition, the feedback message was split into a series of messages discussing individual topics offered while the respondent was still completing the Web-based session, whereas in the summative condition, the entire set of materials/feedback messages was provided at one time at the end of the Web-based session. The actual texts were identical for both conditions. Both subgroups also received a full overview of their advice (equivalent to approximately 7 to 10 A4 pages of text, including pictures and graphics) at the end of a session/measurement, which they could print or save onto their computer. We gave personalized feedback again after 6 months to stimulate participation and to enable us to reassess user satisfaction with the program.

### Questionnaires

#### Drinking Behavior

Weekly alcohol intake was measured by the widely used Dutch 5-item Quantity-Frequency-Variability (QFV) questionnaire [55]. The AUDIT was used to identify problem drinking [49]. Habitual drinking behavior was assessed by the 12-item Self-Report Habit Index (SRHI) questionnaire [56].

#### Psychosocial Determinants

Knowledge regarding the national alcohol guideline was assessed by 1 question: “What do you think is the standard acceptable alcohol amount per day and per week?” with 14 answering options, such as “Two glasses every day is allowed.” A knowledge test was included in the final measurement for all 3 conditions, consisting of 9 questions, such as “How much alcohol is recommended (ie, permitted without having to worry about unfavorable consequences) during pregnancy?” or “How much alcohol does a standard drink contain?”

Attitude was assessed by 6 pros and 6 cons of alcohol intake, such as “Drinking alcohol...allows me to relax” and “...is bad for my health” (1=totally disagree; 5=totally agree; pros alpha=.83, cons alpha=.71).

Social influence was assessed by dividing this concept into norm, modeling, and support. Norm was assessed using the following item “According to people in my immediate environment, I should definitely drink no more than 1 glass (women) or 2 glasses (men) of alcohol a day (=1)” to “I should definitely drink more than 1 glass (women) or 2 glasses (men) of alcohol a day (=5).” Modeling was assessed by asking “How many people in your immediate environment drink no more than 1 glass (women) or 2 glasses (men) of alcohol a day?” (1=nobody; 5=everybody). Support was assessed by including the statement “People in my direct environment support me in my efforts to drink no more than 1 glass (women) or 2 glasses (men) of alcohol a day” (1=no, they don’t support me at all; 4=yes, they support me very much).

Self-efficacy was assessed by 6 items regarding difficult social, emotional, and routine situations, such as “I’m able to meet the alcohol guideline...when I’m at a party,” “...when I feel stressed or nervous,” and “...during a meal” (1=no, definitely not; 2=yes, definitely; alpha=.81).

Preparatory plans were assessed by 4 items, such as “I’m planning to take less money with me when I go out, so I can’t buy a lot of alcoholic drinks” (1=no, definitely not; 5=yes, definitely; alpha=.77).

Coping plans were assessed by 6 items regarding the various risk situations, such as “I’ve made a plan to drink no more than 1 glass (women) or 2 glasses (men) of alcohol when I feel stressed or nervous” (1=totally disagree; 5=totally agree, alpha=.96).

Motivational stage of drinking in accordance with the alcohol guideline was assessed by applying the Transtheoretical Model of Behavior Change [54]. We used 1 item: “Do you intend to drink on no more than 5 days per week and no more than 1 glass (women) or 2 glasses (men) of alcohol a day?” (1=no, I don’t intend to do so; 2=I never thought about it; 3=I thought about it, but I don’t know yet; 4=yes, but not within the next 5 years; yes, 5=within 1-5 years; 6=yes, within 6-12 months, 7=yes, within 3-6 months; 8=yes, within 1-3 months; 9=yes, within a month; 10=yes, and I’m already doing so).

#### Health Status

Six items were used to assess if respondents suffered from diabetes mellitus, angina pectoris, cancer, or high blood pressure or had suffered a stroke or cardiac infarction. Symptoms of depression were assessed by means of the 10-item Center for Epidemiologic Studies Depression Scale (CES-D10) [57].

#### Demographic Information

The following demographic variables were assessed: age, gender (1=male; 2=female), educational level (1=low/no education or primary education; 2=medium/secondary education; 3=high/tertiary education), income (euros per month), employment situation (1=paid employment; 2=no paid employment), marital status, pregnancy/breastfeeding status (1=pregnant/breastfeeding and drinking; 2=n/a), number of children living at home, and native country (1=Germany; 2=other country).

#### Appreciation of the Program

Both experimental subgroups were invited to fill in an evaluation questionnaire to assess the levels of personalization and their appreciation of the intervention. Seven questions were included, such as “The personal advice I received was interesting” (1=no, absolutely not; 5=yes, absolutely).

### Primary Objective

The primary objective was to compare the experimental group (ie, the subgroups who received the computer-tailored feedback strategies) with the control group regarding (1) complying with the alcohol guideline (healthy drinking; yes/no) after 6 months, and (2) mean weekly alcohol consumption (in number of standard drinks) at 6 months after baseline.

### Secondary Objective

The second objective was to compare the 2 computer-tailored feedback strategies (alternating vs summative) in terms of dropout rates, effects on drinking behavior, and appreciation of the program.

### Power Analyses

We estimated the required sample size for both the logistic regression analysis and the linear regression analysis based on the intervention effects of a comparable study by Riper et al [25]. For the logistic regression analysis, a power analysis calculation indicated that a total sample of 180 respondents was needed (after possible attrition) to test for the intervention effect. We calculated the sample size for 2 experimental groups and 1 control group based on a .05 level of significance, a statistical power of 80%, and a 2-sided test. We expected that the compliance with the guideline would be 20% in the intervention groups and 5% in the control group. For the linear regression analysis, a power analysis calculation indicated that a total sample of 254 respondents was needed (after possible attrition) to test for the intervention effect. Again, we calculated the sample size for 2 experimental groups and 1 control group, based on a .05 level of significance, a statistical power of 80%, a 2-sided test, an effect size of 0.30 (when contrasting the 2 intervention groups with the control group), and a correlation of 0.60 between premeasurement and postmeasurement of the outcome variable.

### Statistical Analyses

The data were analyzed using SPSS software, version 19 (IBM Corp, Armonk, NY, USA). To check whether the randomization had been successful in terms of demographics and drinking behavior; linear regression analyses were used for continuous variables and chi-square tests for discrete variables. Descriptive statistics were used for the characteristics of the study sample and the dropout rate within the groups. Logistic regression analyses were performed to determine differences in dropout rates between the study conditions.

The 2 experimental groups together were compared to the control group for drinking behavior. First, effect sizes were calculated based on means and odds ratios (Cohen’s *d*). Effect sizes below 0.30 were considered small, whereas those between 0.30 and 0.80 were considered medium, and those greater than 0.80 were regarded as large [58]. Second, differences in effect between the groups were explored by means of logistic as well as linear regression analyses. The following baseline variables were entered as independent variables in both types of regression analyses using the backward method: condition, gender, age, educational level, employment status, income, country of birth, marital status, having children, pregnancy, disease, CES-D10, number of alcoholic drinks, AUDIT, SRHI, pros, cons, social support, social modeling, social norm, self-efficacy, coping plans, and intention. Preparatory plans were not included in the analyses because not every participant was presented with these items. The dependent variables were (1) meeting the guideline (0=no; 1=yes), and (2) the weekly number of alcoholic drinks after 6 months.

Linear regression analyses were used to determine differences in program evaluation between the 2 experimental subgroups. The dependent variables were the separate items regarding appreciation of the program. Those demographic variables that differed between the study groups were included in the analyses as covariates.

Tests were performed at alpha=.05 for the intervention factor and alpha=.10 for covariates [59]. Analyses were done on data for complete cases only as well as intention-to-treat (ITT) analyses, in which multiple imputation [60] was used to fill in missing values. Missing values were filled using demographics, health status, psychosocial determinants, baseline drinking behavior, drinking behavior after 3 and 6 months, and study condition as predictors. The number of imputations was set at 55. This was done according to the recommendation to create as many imputed datasets as the percentage of cases with missing data [61]. In addition, we also conducted a sensitivity analysis in which the last observation carried forward (LOCF) method was used to fill in missing values.

## Results

### Participation and Attrition

**Figure 2:**
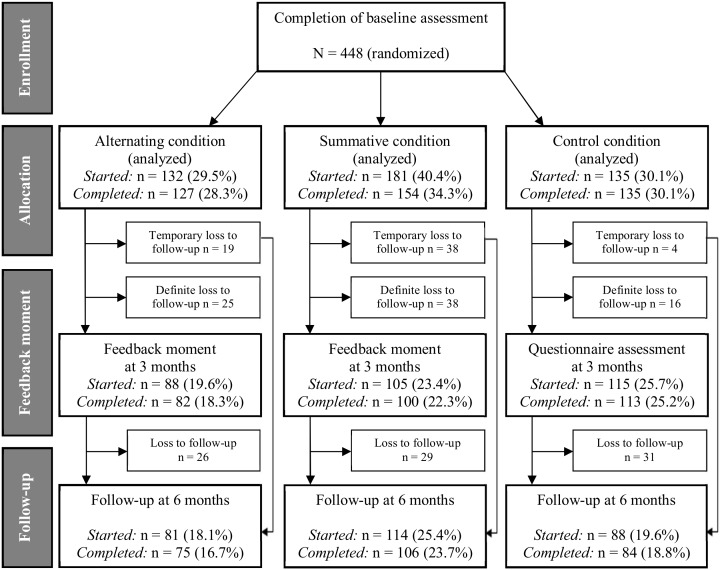
Flowchart of the study sample.

Figure 2 presents a flowchart for the study participants. A total of 1149 participants logged on to the program; 614 did not meet the inclusion criteria and 87 respondents provided incomplete or missing data, resulting in a total sample size of 448 respondents. At the 3-month feedback moment, loss to follow-up was 31.3% (140/448). The dropout rate differed significantly among the 3 conditions (*P*=.001): dropout was significantly lower in the control condition compared to the alternating condition (OR 0.40, 95% CI 0.22-0.73, *P*=.003) and compared to the summative condition (OR 0.35, 95% CI 0.19-0.63, *P*<.001). Moreover, dropout was lower among men (OR 1.48, 95% CI 0.95-2.32, *P*=.08) and among respondents with a high educational level compared to those with a low educational level (OR 0.60, 95% CI 0.36-1.00, *P*=.05) although this did not reach statistical significance. At 6 months, loss to follow-up was 36.8% (165/448) and the dropout rate was distributed equally among the 3 conditions (*P*=.74); however, there was a significant difference in dropout among respondents with different levels of income (*P*=.03); the dropout rate was lower in respondents with the highest income compared to those with the lowest income (OR 0.36, 95% CI 0.15-0.83, *P*=.02).

### Differences in Completion-Rate Between the Experimental Subgroups

During the first session, the dropout rate of the intervention was significantly lower in the alternating condition compared to the summative condition. In the alternating condition, 96.2% (127/132) completed the program whereas in the summative condition, 85.1% (154/181) did so (OR 0.23, 95% CI 0.08-0.60, *P*=.003). However, differences regarding attrition were no longer significant after 3 and 6 months. After 3 months, 62.1% (82/132) of those in the alternating condition and 55.2% (100/181) of those in the summative condition returned to the website and filled in the program completely (OR 0.94, 95% CI 0.57-1.54, *P*=.80). At 6-month follow-up, 56.8% (75/132) of those in the alternating condition and 58.6% (106/181) of those in the summative condition returned to the website and filled in the program completely (OR 1.24, 95% CI 0.77-2.01, *P*=.38).

### Sample Characteristics

Slightly more men than women were included in the study and the mean age of the respondents was approximately 42 years. The average weekly alcohol intake was almost 13 glasses. The baseline demographic characteristics of the study sample are shown in Table 1. Significant differences at the *P*<.10 level were found for the baseline characteristic of income (χ^2^_6_=14.70; *P*=.02) and habitual drinking (beta=0.10, 95% CI 0.00-0.18, *P*=.04).

### Intervention Effects

**Figure 3:**
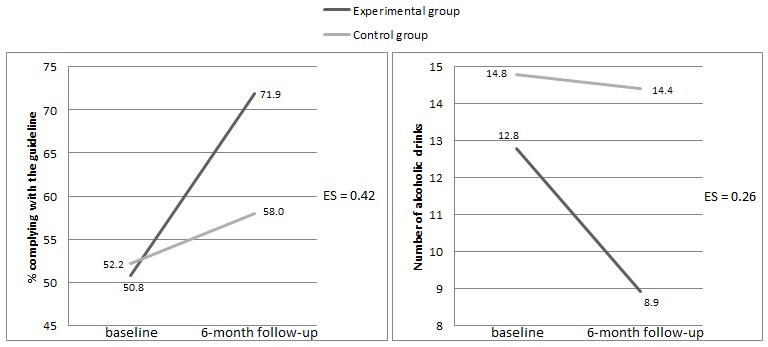
Differences and effect sizes (ES) regarding compliance with the alcohol guideline among complete cases (n=197) and number of alcoholic drinks per week between the experimental group and the control group at baseline and after 6 months.

The number of respondents who complied with the alcohol guideline rose after 6 months (Figure 3). The percentage of respondents complying with the guideline increased by 21.1% in the experimental group and by 5.8% in the control group. The number of alcoholic drinks per week among the study population also decreased during the intervention period (Figure 3). The experimental group reduced their mean weekly alcohol intake by 3.9 drinks (SD 9.96) compared to 0.4 drinks (SD 19.54) in the control group.

As shown in Table 2, the results of the logistic regression analysis showed that the intervention was effective in achieving a low-risk drinking status according to the guideline among complete cases (OR 2.65; *P*=.02). However, different results were found when using ITT analyses. After applying multiple imputations, no intervention effect was found among the study sample (OR 1.11; *P*=.72). Results of the sensitivity analysis using LOCF can be found in Multimedia Appendix 1.

The linear regression analysis among complete cases (see Table 3) found an effect for the intervention in lowering the weekly number of alcoholic beverages in the experimental group, but this did not reach statistical significance (beta=−0.12, 95% CI −7.96 to 0.03, *P*=.05). After applying multiple imputations, no intervention effect was found (B=−1.15, 95% CI −4.02 to 1.72, *P*=.43).

### Differences Between the Two Experimental Subgroups

A comparison between the 2 experimental subgroups (n=128) regarding compliance with the guideline (OR 0.41, 95% CI 0.13-1.36, *P*=.15) and weekly alcohol intake (beta=−0.03, 95% CI −3.14 to 2.11, *P*=.70) showed no differences in effect. Comparable results were found after multiple imputations of missing values. There were neither differences regarding achievement of low-risk drinking status according to the guideline (OR 0.72, 95% CI 0.38-1.37, *P*=.31) nor regarding the weekly number of alcoholic beverages after 6 months (B=−0.11, 95% CI −2.89 to 2.68, *P*=.94) between the 2 experimental subgroups.

### Differences in Appreciation of the Program

In general, the intervention was evaluated positively by both experimental subgroups (Table 4). At baseline, respondents of the alternating condition reported that they had read more of the advice compared to respondents of the summative condition; however, this difference did not meet statistical significance (*P*=.07). After 6 months, this difference was not apparent. At the 6-month follow-up measurement, the advice was perceived as more informative among the summative condition compared to the alternating condition, although this did not meet statistical significance (*P*=.08).

**Table 1:** Demographics, health status, and drinking behavior of the study sample at baseline.

Variable		Total N=448	Alternating condition n=132	Summative condition n=181	Control condition n=135
Age (18-69 years), mean (SD)		41.72 (15.74)	42.23 (15.06)^a^	41.41 (16.16)	41.62 (15.92)
**Gender, n (%)**					
	Male	253 (56.5)	69 (52.3)	104 (57.5)	80 (59.3)
	Female	195 (43.5)	63 (47.7)	77 (42.5)	55 (40.7)
**Education, n (%)**					
	Low	177 (42.0)	61 (47.3)	61 (38.9)	55 (40.7)
	Medium	101 (24.0)	25 (19.4)	40 (25.5)	36 (26.7)
	High	143 (34.0)	43 (33.3)	56 (35.7)	44 (32.6)
**Income per month, n (%)**					
	<€1000	61 (13.6)	11 (8.3)	24 (13.3)	26 (19.3)
	€1001-€2000	106 (23.7)	41 (31.1)	30 (16.6)	35(25.9)
	€2001-€4000	135 (30.1)	34 (25.8)	55 (30.4)	46 (34.1)
	>€4000	43 (9.6)	19 (14.4)	12 (6.6)	12 (8.9)
	Not reported	103 (23.0)	27 (20.5)	60 (33.1)	16 (11.9)
**Employment situation, n (%)**					
	Job (paid employment)	269 (65.3)	89 (71.8)	97 (63.4)	83 (61.5)
	No job	143 (34.7)	35 (28.2)	56 (36.6)	52 (38.5)
**Marital status, n (%)**					
	Married	170 (40.4)	55 (42.6)	62 (39.5)	53 (39.3)
	Living together	67 (14.9)	26 (20.2)	28 (17.8)	13 (9.6)
	In relationship, but not living together	51 (12.1)	12 (9.3)	22 (14.0)	17 (12.6)
	Single/unmarried	90 (21.4)	22 (17.1)	35 (22.3)	33 (24.4)
	Divorced	31 (7.4)	9 (7.0)	6 (3.8)	16 (11.9)
	Widowed	12 (2.9)	5(3.9)	4 (2.5)	3 (2.2)
**Children, n (%)**					
	No	226 (50.4)	58 (43.9)	100 (55.2)	68 (50.4)
	Yes, but no longer living at home	93 (20.8)	26 (19.7)	33 (18.2)	34 (25.2)
	Yes, living at home >18 years	34 (7.6)	14 (10.6)	11 (6.1)	9 (6.7)
	Yes, living at home <18 years	95 (21.2)	34 (25.8)	37 (20.4)	24 (17.8)
**Native country, n (%)**					
	Germany	409 (97.1)	126 (97.7)	152 (96.8)	131 (97.0)
	Other	12 (2.9)	3 (2.3)	5 (3.2)	4 (3.0)
**Symptoms of depression**					
	CES-D10, mean (SD)^b^	8.20 (5.05)	8.08 (5.46)	8.38 (5.05)	8.11 (4.68)
	Score of ≥11, n (%)	120 (28.8)	39 (30.7)	44 (28.6)	37 (27.4)
**Diseases, n (%)**					
	Diabetes mellitus	21 (4.7)	7 (5.2)	9 (5.0)	5 (3.7)
	Stroke	8 (1.8)	1 (0.7)	3 (1.7)	4 (3.0)
	Cardiac infarction	7 (1.6)	1 (0.7)	3 (1.7)	3 (2.2)
	Angina pectoris	9 (2.0)	2 (1.5)	4 (2.2)	3 (2.2)
	Cancer	6 (1.3)	0 (0.0)	4 (2.2)	2 (1.5)
	High blood pressure	95 (21.1)	26 (19.3)	41 (22.7)	28 (20.7)
	One or more diseases	128 (28.6)	35 (26.5)	55 (30.4)	38 (28.1)
**Alcohol**					
	Nonadherence to guideline, n (%)	221 (51.4)	63 (47.7)	85 (49.7)	73 (54.9)
	Weekly alcohol intake (standard units), mean (SD)^c^	12.94 (11.24)	12.53 (10.99)	11.86 (9.70)	14.73 (13.05)
	Pregnant/ breastfeeding and drinking, n (%)	31 (6.9)	8 (6.1)	14 (7.7)	9 (6.7)
	AUDIT (score ≥8), n (%)	351 (80.0)	102 (77.3)	141 (79.2)	108 (81.2)
	Habit (SRHI-12), mean (SD)^d^	2.11 (0.82)	1.98 (0.79)	2.15 (0.79)	2.19 (0.86)

^a^Age range 18-68 years.

^b^Ranges for total, alternating, summative, and control were 0.00-28.00, 0.00-28.00, 0.00-28.00, and 0.00-22.00, respectively.

^c^Ranges for total, alternating, summative, and control were 0.00-86.00, 0.00-70.00, 0.00-66.00, and 0.50-86.00, respectively.

^d^Ranges for total, alternating, summative, and control were 1.00-4.83, 1.00-4.83, 1.00-4.50, and 1.00-4.33, respectively.

**Table 2:** Results of the logistic regression analysis (backward method) with guideline status (0=not complying; 1=complying) after 6 months as dependent variable among complete cases (CC, n=197) and after applying multiple imputations (MI, n=448).

Variable^a^	Guideline status (CC)			Guideline status (MI)		
	OR	*P*	95% CI	OR	*P*	95% CI
Condition	2.65	.02	1.14, 6.16	1.11	.72	0.63, 1.98
Guideline status	—	—	—	2.91	<.001	1.63, 5.18
Weekly alcohol intake	0.88	<.001	0.84, 0.93	0.96	.04	0.93, 1.00
Habit	0.23	<.001	0.12, 0.42	0.46	<.001	0.31, 0.70
AUDIT	0.40	.07	0.15, 1.09	—	—	—
Age	0.96	.007	0.94, 0.99	—	—	—
Self-efficacy	0.47	.03	0.24, 0.94	0.62	.045	0.39, 0.99
Intention	0.88	.03	0.78, 0.98	—	—	—
*R* ^*2*^	0.52			0.32		

^a^Assessed at baseline.

**Table 3:** Results of the linear regression analysis (backward method) with the number of alcoholic drinks after 6 months as dependent variable among complete cases (CC, n=197) and after applying multiple imputations (MI, n=448).

Variable^a^	Number of drinks (CC)			Number of drinks (MI)		
	β	*P*	CI	B	*P*	CI
Condition	−0.12	.05	−7.96, 0.03	−1.15	.43	−4.02, 1.72
Weekly alcohol intake	0.49	<.001	0.52, 0.86	0.61	<.001	0.47, 0.75
Habit	0.18	.01	0.77, 60.36	2.65	.01	0.67, 4.64
Native country	0.10	.09	−1.21, 18.11	—	—	—
Social norm	—	—	—	−1.20	.049	−2.39, −0.01
Self-efficacy	0.14	.049	0.01, 6.17	2.08	.06	−0.12, 4.29
*R* ^*2*^	0.33			0.29		

^a^Assessed at baseline.

**Table 4:** Differences between the 2 experimental subgroups (alternating condition: n=59; summative condition: n=72) regarding the evaluation items about appreciation of the program.

Items	Baseline				6-month follow-up			
	Alternating condition	Summative condition	β	*P*	Alternating condition	Summative condition	β	*P*
	Mean (SD)	Mean (SD)			Mean (SD)	Mean (SD)		
Evaluation mark^a^	11.31 (3.22)	11.51 (3.29)	0.03	.72	12.05 (2.99)	12.08 (3.24)	0.01	.95
I have read all pieces of advice^b^	4.34 (0.78)	4.07 (0.89)	−0.16	.07	3.95 (0.92)	3.92 (0.88)	−0.02	.84
The advice was interesting^b^	4.31 (0.90)	4.31 (0.82)	0.00	.99	4.32 (0.88)	4.36 (0.74)	0.01	.89
The advice was credible^b^	4.56 (0.68)	4.40 (0.80)	−0.11	.24	4.46 (0.84)	4.54 (0.69)	0.06	.53
The advice was informative^b^	4.46 (0.90)	4.39 (0.78)	−0.04	.64	4.32 (0.92)	4.57 (0.71)	0.15	.08
The advice was clear^b^	4.53 (0.73)	4.44 (0.73)	−0.06	.53	4.34 (0.86)	4.50 (0.73)	0.13	.14
The advice helps me to drink less alcohol^b^	3.41 (1.19)	3.47 (1.07)	0.03	.74	3.75 (1.09)	3.68 (1.07)	0.01	.93

^a^Scores: 0 (very bad)-15 (excellent).

^b^Scores: 1 (no, absolutely not)-5 (yes, absolutely).

## Discussion

### Principal Findings

This study used a randomized controlled trial to determine the effectiveness of a Web-based tailored alcohol intervention, and to compare the effects of 2 tailoring strategies in terms of drinking behavior change, dropout rates, and appreciation. The experimental group and the control group both decreased their alcohol consumption, but the effects for our primary outcomes were greater in the experimental group. Complete case analyses as well as ITT analyses were performed. Inconsistent results were found. First of all, only among complete cases intervention effects were identified in terms of meeting the alcohol guidelines. Second, the experimental group reduced their weekly alcohol intake by a greater amount than the control group, although this effect did not reach statistical significance when performing complete case analyses and multiple imputations.

The results of this study partly confirm that Web-based tailored self-help interventions can be an effective tool in decreasing alcohol consumption and encouraging low-risk drinking in adults [25,32]. It is noteworthy that the control group also achieved a small reduction in alcohol intake and an increase in the percentage of respondents adhering to the guideline. This finding is in-line with previous studies, which also found effects in control groups regarding alcohol intake (eg, [26]) as well as regarding other lifestyle behaviors, such as physical activity [62]. Assessment alone can already have significant effects on drinking. The act of completing an assessment questionnaire may have induced the participants in the control group to monitor and reflect on their own behavior, leading to a decrease in consumption [34].

Regarding our secondary goal—comparing an alternating and a summative tailoring strategy—we found no difference between the 2 strategies for changes in alcohol use. These results were the same among complete cases and ITT. Because both experimental subgroups ultimately received the same advice, the timing of the message delivery does not appear to have influenced behavioral impact. The attrition rates of our intervention show that more respondents in the alternating group completed the intervention at baseline. These respondents may have felt rewarded by receiving the advice in-between answering the questions, and this strategy may have made the program more attractive. This was partly confirmed by the program evaluation because the alternating group indicated having read more pieces of the personal advice. At 6-month follow-up, however, the dropout rate no longer differed between the 2 experimental subgroups. The appreciation of the program was also comparable between these 2 groups. Respondents who did not revisit the program after 6 months were those who evaluated the program more negatively at baseline, implying selective dropout.

### Strengths and Limitations

Our study was characterized by some strengths. First of all, our intervention program was theory-based. The intervention satisfied the 5 basic criteria (ie, the 5 A’s) of the HBC-I [39,63] in addition to providing other essential tailoring elements. The respondents’ answers to a number of questionnaires were used to give advice about the risks of heavy drinking and about the need to change their drinking behavior; we assessed various possible predictors of behavioral change, such as attitude, social influence, self-efficacy, and planning; we assisted respondents by giving personal advice on the various psychosocial variables, including support and understanding, as well as personal information regarding relapse prevention (anticipatory guidance); and we arranged follow-up sessions. In previous research, extensive use of theory, including the Theory of Planned Behavior [51], has been associated with considerable effects on health-related behavior [64]. Our program was based on the I-Change model, which consists of the Theory of Planned Behavior constructs supplemented by concepts such as awareness factors and action planning strategies. The latter, in particular, is associated with increased behavioral effects, as has been demonstrated in general [65,66] as well as specifically for computer-tailored programs [67]. Our intervention program used multiple tailoring by offering 3 feedback moments. A multisession program is likely to be more effective than a single-session program [68-70]. Further research should explore the optimal number of feedback moments as well as the optimal time lag between the different sessions. To our knowledge, this is the first study to compare 2 different tailoring strategies (ie, alternating vs summative) in terms of effectiveness, dropout, and appreciation. Finally, few studies have tested a Web-based tailored alcohol intervention among adults in the general population [71].

Our study was also subject to some limitations. First, our findings were based on self-reports, which may have led to recall bias. Previous research has shown that quantity-frequency measures, such as those we used in this study, are likely to result in greater underestimation than daily diaries [72]. However, because we used the same questions at all measurement moments, this may have not influenced our data indicating changes in behavior, and thus the effectiveness of the intervention. In any case, forgetting seems to be a potent source of underestimation in surveys regarding alcoholic drinks [55]. Second, all respondents were recruited through an online panel and received an incentive for their participation, which might mean that some of them were not motivated to change their drinking behavior and/or that they took part in this study simply to receive the incentive. Third, our study had a moderate-sized sample and a high attrition rate (approximately 41%) as well as missing values on some baseline data (approximately 26%). Based on the power analyses, the number of participants was sufficient for executing logistic regression analyses; however, for the linear regression analysis, our sample size was too small. Therefore, the effect among complete cases might have not reached statistical significance. However, the effect was found in the expected direction. Although our data still yielded statistically significant effects among the complete cases, it may be that a selective group (ie, a very motivated group) completed the intervention program. This implies that we have to interpret the results regarding the intervention effects of this subgroup carefully. However, further support was obtained in a sensitivity analysis employing LOCF. Thus, data analysis with complete cases and LOCF methods showed statistically significant intervention effects in reaching a low-risk drinking status and an effect of the intervention in decreasing the weekly amount of alcohol intake, although without reaching statistical significance. Analyses with multiple imputation methods did not confirm these findings. Although multiple imputation methods are regarded as the most preferred technique to handle missing data [73], our analyses showed remarkable differences in outcomes. Moreover, the use of multiple imputation techniques may result in unreliable estimates when the number of missing values is high [74], as is the case in our study. Consequently, more research is needed to outline the conditions that yield these differences between the approaches. Additionally, when applying multiple imputation, the strategies how this technique is used should be clearly documented because multiple imputation techniques require certain procedures and rules [61,74-75]. At this moment, there seems to be no consensus about the number of imputed datasets needed [61,73-76]. Other shortcomings of multiple imputation are that the results may strongly depend on the imputation model that is created [74,77] and that different multiple imputation programs seem to show different results [73]. Finally, although we had a follow-up measurement 6 months after baseline, the long-term impact of Web-based tailored interventions still remains unclear and requires further research.

### Conclusions

Tailored feedback delivered via the Internet can be an effective way to reduce alcohol intake among adults, at least among a subgroup that revisited the program. However, the results of complete case analyses were inconsistent with the findings of ITT analyses when using the multiple imputation technique. Among our Internet panel, there were no indications that an alternating or a summative tailoring strategy works better in reducing alcohol intake by means of eHealth programs. Nevertheless, lower attrition rates during the first visit indicate that the version of the intervention with alternating questions and advice may be preferred.

## Multimedia Appendix 1

Results of regression analyses after filling missing values with the last observation.

## Multimedia Appendix 2

CONSORT-EHEALTH checklist V1.6.2 [78].

## Abbreviations

AUDIT: Alcohol Use Disorders Identification Test
ISRCTN: International Standard Randomized Controlled Trial Number
ITT: intention-to-treat
LOCF: last observation carried forward
QFV: Quantity-Frequency-Variability
SRHI: Self-Report Habit Index
