# Supplementary material for: Effects of a Web-Based Tailored Intervention to Reduce Alcohol Consumption in Adults: Randomized Controlled Trial
Source: J Med Internet Res. 2013 Sep 17;15(9):e206. doi: 10.2196/jmir.2568 (PMC3785997; doi:10.2196/jmir.2568)
Supplement: Supplementary file 1 [file jmir_v15i9e206_app1.pdf]

Table 5: *Results of the logistic regression analysis (backward method) with guideline status (not complying = 0; complying = 1) after 6 months as dependent variable and results of the linear regression analysis (backward method) with the number of alcoholic drinks after 6 months as dependent variable after filling missing values with the last observation (LOCF; n = 321)*

	**Guideline status (LOCF)**			**Number of drinks (LOCF)**		
**Variable** [1]	**OR**	***P***	**CI**	**β**	***P***	**CI**
Condition	2.29	.009	1.23-4.26	-.07	.09	-4.80-.32
Guideline status	4.88	<.001	2.32-10.27	---	---	---
Weekly alcohol intake	.93	.002	.89-.97	.58	<.001	.61-.84
Habit	.34	<.001	.22-.53	.14	.006	.71-4.32
Age	.98	.08	.96-1.00	---	---	---
Social norm	---	---	---	-.08	.07	-2.04-.09
Self-efficacy	.48	.003	.29-.77	.10	.045	.05-3.97
R^2	*.49*			*.43*		

[1] Assessed at baseline
