# Supplementary material for: Effects of a Web-Based Tailored Intervention to Reduce Alcohol Consumption in Adults: Randomized Controlled Trial
Source: J Med Internet Res. 2013 Sep 17;15(9):e206. doi: 10.2196/jmir.2568 (PMC3785997; doi:10.2196/jmir.2568)
Supplement: Supplementary file 2 [file jmir_v15i9e206_app2.pdf]

## CONSORT-EHEALTH Checklist V1.6.2 Report

**(based on CONSORT-EHEALTH V1.6), available at [http://tinyurl.com/consort-ehealth-v1-6].**

2568

**Date completed**

2/6/2013 9:18:34

**by**

Daniela Schulz

Effects of a web-based tailored intervention to reduce alcohol consumption by adults: A randomized controlled trial

### TITLE

**1a-i) Identify the mode of delivery in the title**

YES: "[...] web-based tailored intervention [...]"

**1a-ii) Non-web-based components or important co-interventions in title**

n/a

**1a-iii) Primary condition or target group in the title**

YES: "[...] by adults [...]"

### ABSTRACT

**1b-i) Key features/functionalities/components of the intervention and comparator in the METHODS section of the ABSTRACT**

YES: "Drinking behaviour, health status, motivational determinants (e.g., attitude, social influence, self-efficacy, preparatory plans, coping plans and motivational stage) and demographics were assessed among participants [...]"

**1b-ii) Level of human involvement in the METHODS section of the ABSTRACT**

**1b-iii) Open vs. closed, web-based (self-assessment) vs. face-to-face assessments in the METHODS section of the ABSTRACT**

"[...] recruited via an online access panel."

**1b-iv) RESULTS section in abstract must contain use data**

"[...] Around 21.1% of those respondents of the experimental group who did not comply with the German national guideline for low-risk drinking at baseline did meet this guideline after six months, compared to 5.8% in the control group (ES = .42; OR = 2.65; P = .02). The decrease by 3.9 drinks per week in the experimental group was almost significantly greater than the 0.4 drinks decrease in the control group (ES = .26; β = -.12; P = .052). Separate analyses of the two experimental subgroups showed no differences in intervention effects. The drop-out rate during the first visit to the intervention website was significantly lower in the alternating condition than in the summative condition (OR = .23; P = .003). Programme appreciation was comparable for the two experimental groups."

The number of participants is already mentioned in the method section (N=448).

**1b-v) CONCLUSIONS/DISCUSSION in abstract for negative trials**

### INTRODUCTION

**2a-i) Problem and the type of system/solution**

" [...] The high prevalence of unhealthy drinkers and the low number of them who seek help underline the need for easily accessible and low-threshold interventions to encourage people to reduce their alcohol intake.

Web-based tailored interventions, in which information is adapted to the user's individual characteristics and needs in order to give them personally appropriate advice [12,13], have proved an effective tool to improve health-related behaviours. [...] To date, several studies of web-based tailored alcohol interventions have been published, but randomized controlled trials among the general adult population using tailored alcohol self-help programmes have been scarce [25-31]."

**2a-ii) Scientific background, rationale: What is known about the (type of) system**

"[...] Little research has been done to assess what elements work well in tailored interventions. [...] Although Internet-based programmes have the potential to reach large numbers of people, various studies have pointed out that the actual use may be limited and that high rates of attrition are common [40-45]. In order to prevent early drop-out, and thus to increase the effectiveness of a programme, two different strategies could be used to hold respondents' attention in online interventions. In the first strategy, questions and advice are given alternately, so that the respondents are rewarded while they are still filling in the questionnaires and are thereby motivated to continue. Such alternation might also enhance the attractiveness of the programme. In the second strategy, advice is given in a more traditional way, at the end of the session (i.e. after the last questionnaire has been completed)."

### METHODS

**3a) CONSORT: Description of trial design (such as parallel, factorial) including allocation ratio**

"The objective of our study was twofold. First, we explored the overall effectiveness of a three-session, web-based tailored alcohol intervention for unhealthy drinkers in the general adult population. Second, we compared the drop-out rate, effectiveness and user satisfaction of two kinds of feedback strategy (alternating versus summative)."

**3b) CONSORT: Important changes to methods after trial commencement (such as eligibility criteria), with reasons**

We sent out up to two reminders to increase the response.

**3b-i) Bug fixes, Downtimes, Content Changes**

**4a) CONSORT: Eligibility criteria for participants**

"The following inclusion criteria were established for this study: being a panel member of respondi; having computer / Internet literacy; having sufficient command of German; being at least 18 years old; and having an unhealthy drinking pattern, which was defined as (1) not complying with the guideline recommending no more than 1 glass (women) / 2 glasses (men) of alcohol a day; (2) drinking on more than 5 days of the week; (3) having a score higher than 7 on the AUDIT [47]; or (4) currently trying to become pregnant, drinking alcohol while pregnant or breastfeeding (in relation to pregnancy) (for women) or trying to get one's partner pregnant (for men)."

**4a-i) Computer / Internet literacy**

[...] having computer / Internet literacy [...]

**4a-ii) Open vs. closed, web-based vs. face-to-face assessments:**

"The intervention, focusing on unhealthy drinkers in the general population, was conducted online in Germany in 2010-2011. Adult participants were recruited via an online access panel (respondi AG Cologne). The sample received an e-mail containing a link to either the intervention website (experimental group) or a web-based alcohol questionnaire (control group). Two reminder messages in the form of e-mails were sent to individuals of the sample who did not respond to the first invitation."

**4a-iii) Information giving during recruitment**

**4b) CONSORT: Settings and locations where the data were collected**
"The intervention, focusing on unhealthy drinkers in the general population, was conducted online in Germany in 2010-2011. Adult participants were recruited via an online access panel (respondi AG Cologne)."
**4b-i) Report if outcomes were (self-)assessed through online questionnaires**
Outcomes were self-assessed through online questionnaires
**4b-ii) Report how institutional affiliations are displayed**

**5) CONSORT: Describe the interventions for each group with sufficient details to allow replication, including how and when they were actually administered**
**5-i) Mention names, credential, affiliations of the developers, sponsors, and owners**

**5-ii) Describe the history/development process**
We had executed a pilot study in the Netherlands.
**5-iii) Revisions and updating**
n/a - no major changes were made
**5-iv) Quality assurance methods**

**5-v) Ensure replicability by publishing the source code, and/or providing screenshots/screen-capture video, and/or providing flowcharts of the algorithms used**
We included a screen shot in the manuscript.
**5-vi) Digital preservation**

**5-vii) Access**
Panel members of the online access panel respondi (Cologne) were invited to take part in the study. "The sample received an e-mail containing a link to either the intervention website (experimental group) or a web-based alcohol questionnaire (control group)." Participation was free of charge. "Incentives, in the form of bonus points which respondents could exchange for cash, for a gift voucher or for a charitable donation, were given to respondents who filled in the questionnaires completely."
**5-viii) Mode of delivery, features/functionalities/components of the intervention and comparator, and the theoretical framework**
"The theoretical framework for the development of the intervention was the I-Change model [48]."

In the tailored advice, we made use of texts, graphs and pictures.

"Conditions
All study groups received identical questionnaires. After completing the third measurement, the waiting list control group were given the link to the intervention website where these respondents also had the possibility to receive personalised advice. The experimental condition was divided into two experimental subgroups. The intervention website for these two subgroups offered the same feedback messages. At all three feedback moments (at baseline, after three months and after six months), one experimental subgroup received questions and personal advice alternately (alternating condition) whereas the other experimental subgroup were given one complete set of personal advice after having answered all the questions (summative condition). In other words, in the alternating condition, the feedback message was split up into a series of messages discussing individual topics, which were offered while the respondent was still completing the web-based session, while in the summative condition, a single set of materials / feedback messages was provided in one go at the end of the web-based session. The actual texts were identical for both conditions. Both subgroups also received a full overview of their pieces of advice (equivalent to approximately seven to ten A4 pages of text, including pictures and graphics) at the end of a session / measurement, which they could print or save on their computer. We gave personalised feedback again after six months to stimulate participation and to enable us to assess user satisfaction with the programme again."
**5-ix) Describe use parameters**

**5-x) Clarify the level of human involvement**

**5-xi) Report any prompts/reminders used**
"Two reminder messages in the form of e-mails were sent to individuals of the sample who did not respond to the first invitation."
**5-xii) Describe any co-interventions (incl. training/support)**
Our intervention was a stand-alone intervention.
**6a) CONSORT: Completely defined pre-specified primary and secondary outcome measures, including how and when they were assessed**
Participants had the possibility to e-mail us with questions, feedback and (technical) problems.
**6a-i) Online questionnaires: describe if they were validated for online use and apply CHERRIES items to describe how the questionnaires were designed/deployed**

**6a-ii) Describe whether and how "use" (including intensity of use/dosage) was defined/measured/monitored**
In our program, we have the possibility to see the time respondents spent on the web-based program and on the different sites within the program.
**6a-iii) Describe whether, how, and when qualitative feedback from participants was obtained**
Participants had the possibility to e-mail us with questions, feedback and (technical) problems.
**6b) CONSORT: Any changes to trial outcomes after the trial commenced, with reasons**
n/a
**7a) CONSORT: How sample size was determined**
**7a-i) Describe whether and how expected attrition was taken into account when calculating the sample size**

**7b) CONSORT: When applicable, explanation of any interim analyses and stopping guidelines**
n/a
**8a) CONSORT: Method used to generate the random allocation sequence**
Respondents were randomized into the different study groups by the online access panel.
**8b) CONSORT: Type of randomisation; details of any restriction (such as blocking and block size)**
Randomization took place at the individual level.

**9) CONSORT: Mechanism used to implement the random allocation sequence (such as sequentially numbered containers), describing any steps taken to conceal the sequence until interventions were assigned**

Randomization took place at the individual level.

**10) CONSORT: Who generated the random allocation sequence, who enrolled participants, and who assigned participants to interventions**

The randomization process was done by the online access panel.

**11a) CONSORT: Blinding - If done, who was blinded after assignment to interventions (for example, participants, care providers, those assessing outcomes) and how**

**11a-i) Specify who was blinded, and who wasn't**

The respondents were blinded.

**11a-ii) Discuss e.g., whether participants knew which intervention was the "intervention of interest" and which one was the "comparator"**

Since panel members of the online access panel most of the times are invited to take part in assessment-only studies, we guess that the control condition did not know that they did not receive the intervention of interest.

**11b) CONSORT: If relevant, description of the similarity of interventions**

n/a

**12a) CONSORT: Statistical methods used to compare groups for primary and secondary outcomes**

"The data were analysed using SPSS software, version 19. To check whether the randomisation had been successful in terms of demographics and drinking behaviour, linear regression analyses were used for continuous variables and Chi-square tests for discrete variables. Descriptive statistics were used for the characteristics of the study sample and the drop-out rate within the groups. Logistic regression analyses were performed to determine differences in drop-out rates between the study conditions.

The two experimental groups together were compared to the control group in terms of drinking behaviour. First, effect sizes (ES) were calculated based on means and odds ratios (Cohen's d). Effect sizes below 0.30 were considered small, while those between 0.30 and 0.80 were considered medium, and those larger than 0.80 were regarded as large [53]. Second, differences in effect between the groups were explored by means of logistic as well as linear regression analyses. The following baseline variables were entered as independent variables in both types of regression analyses, using the backward method: condition, gender, age, educational level, employment status, income, country of birth, marital status, having children, pregnancy, disease, CES-D10, number of alcoholic drinks, AUDIT, SRHI, pros, cons, social support, social modelling, social norm, self-efficacy, coping plans and intention. Preparatory plans were not included in the analyses since not every participant was presented with these items. The dependent variables were (1) meeting the guideline (no=0; yes=1) and (2) the number of alcoholic drinks after six months.

Linear regression analyses were used to determine differences in programme evaluation between the two experimental subgroups. The dependent variables were the separate items regarding appreciation of the programme. Those demographic variables that differed between the study groups were included in the analyses as covariates.

Tests were performed at $\alpha = .05$ for the intervention factor and $\alpha = .10$ for covariates [54]."

**12a-i) Imputation techniques to deal with attrition / missing values**

"To test the robustness of the results obtained for completers-only (CO), we also conducted a sensitivity analysis where the last observation carried forward (LOCF) method was used to fill in missing values."

**12b) CONSORT: Methods for additional analyses, such as subgroup analyses and adjusted analyses**

"Linear regression analyses were used to determine differences in programme evaluation between the two experimental subgroups."

## RESULTS

**13a) CONSORT: For each group, the numbers of participants who were randomly assigned, received intended treatment, and were analysed for the primary outcome**

We added an attrition diagram in which these results are shown.

**13b) CONSORT: For each group, losses and exclusions after randomisation, together with reasons**

We added an attrition diagram in which these results are shown.

**13b-i) Attrition diagram**

We added an attrition diagram in which these results are shown. We do not know the reasons for drop-out.

**14a) CONSORT: Dates defining the periods of recruitment and follow-up**

"[...] with a follow-up measurement after six months. The intervention, focusing on unhealthy drinkers in the general population, was conducted online in Germany in 2010-2011."

**14a-i) Indicate if critical "secular events" fell into the study period**

**14b) CONSORT: Why the trial ended or was stopped (early)**

n/a

**15) CONSORT: A table showing baseline demographic and clinical characteristics for each group**

See Table 1: Demographics, health status and drinking behaviour of the study sample at baseline

**15-i) Report demographics associated with digital divide issues**

See Table 1.

**16a) CONSORT: For each group, number of participants (denominator) included in each analysis and whether the analysis was by original assigned groups**

**16-i) Report multiple "denominators" and provide definitions**

See our flow-chart (Figure 2). Moreover, we added the n's in the description/title of the tables reporting the results.

**16-ii) Primary analysis should be intent-to-treat**

**17a) CONSORT: For each primary and secondary outcome, results for each group, and the estimated effect size and its precision (such as 95% confidence interval)**

See Table 2, Figure 3, and Table 3.

**17a-i) Presentation of process outcomes such as metrics of use and intensity of use**

**17b) CONSORT: For binary outcomes, presentation of both absolute and relative effect sizes is recommended**

See Table 2, Figure 3, and Table 3.

**18) CONSORT: Results of any other analyses performed, including subgroup analyses and adjusted analyses, distinguishing pre-specified from exploratory**

We also compared the effects of the intervention between our two experimental subgroups (regarding drinking behaviour change and appreciation of the program).

**18-i) Subgroup analysis of comparing only users**

We also included complete cases analyses.

**19) CONSORT: All important harms or unintended effects in each group**
No intended harms were reported.
**19-i) Include privacy breaches, technical problems**
It might be that technical problems occured. This might have happened in all study groups.
**19-ii) Include qualitative feedback from participants or observations from staff/researchers**

## DISCUSSION

**20) CONSORT: Trial limitations, addressing sources of potential bias, imprecision, multiplicity of analyses**
**20-i) Typical limitations in ehealth trials**
"First, our findings were based on self-reports, which may have led to recall bias. Previous research has shown that quantity–frequency measures – like those we used in this study – are likely to result in greater underestimation than daily diaries [65]. [...] Second, our study had a moderate-sized sample and a high attrition rate (40.8%). [...] Third, all respondents were recruited through an online panel and received an incentive for their participation, which might mean that some of them were not motivated to change their drinking behaviour and / or that they took part in this study simply to receive the incentive."
**21) CONSORT: Generalisability (external validity, applicability) of the trial findings**
**21-i) Generalizability to other populations**

**21-ii) Discuss if there were elements in the RCT that would be different in a routine application setting**

**22) CONSORT: Interpretation consistent with results, balancing benefits and harms, and considering other relevant evidence**
**22-i) Restate study questions and summarize the answers suggested by the data, starting with primary outcomes and process outcomes (use)**
"This study used a randomized controlled trial to determine the effectiveness of a web-based tailored alcohol intervention, and to compare the effects of two tailoring strategies in terms of drinking behaviour change, drop-out rates and appreciation. The experimental group and the control group both decreased their alcohol consumption, but the effects in terms of our primary outcomes were greater in the experimental group. First of all, intervention effects were identified in terms of meeting the alcohol guidelines. Second, the experimental group reduced their weekly alcohol intake by an almost significantly greater amount than the control group. [...] Regarding our secondary goal – comparing an alternating and a summative tailoring strategy – we found no difference between the two strategies as regards changes in alcohol use. [...] The attrition rates of our intervention show that more respondents in the alternating group completed the intervention at baseline. [...] The appreciation of the programme was also comparable among these two groups."
**22-ii) Highlight unanswered new questions, suggest future research**
"[...]although we had a follow-up measurement six months after baseline, the long-term impact of web-based tailored interventions still remains unclear and requires further research."

## Other information

**23) CONSORT:  Registration number and name of trial registry**
ISRCTN91623132
**24) CONSORT: Where the full trial protocol can be accessed, if available**
n/a
**25) CONSORT: Sources of funding and other support (such as supply of drugs), role of funders**
"This study was funded by the CAPHRI School for Public Health and Primary Care. Intervention development and data analysis took place at Maastricht University. Data collection was done in collaboration with respondi AG Cologne. The authors wish to thank the project partners at respondi AG for their assistance in implementing the intervention and recruiting the participants."
**X26-i) Comment on ethics committee approval**

**x26-ii) Outline informed consent procedures**

**X26-iii) Safety and security procedures**

**X27-i) State the relation of the study team towards the system being evaluated**
"Hein de Vries is scientific director of Vision2Health, a company that licenses evidence-based innovative computer-tailored health communication tools. No other authors reported any conflicts of interest."
